# Efficacy of Pulsed Radiofrequency on Cervical 2-3 Posterior Medial Branches in Treating Chronic Migraine: A Randomized, Controlled, and Double-Blind Trial

**DOI:** 10.1155/2015/690856

**Published:** 2015-06-11

**Authors:** Yuecheng Yang, Xuehua Huang, Yinghui Fan, Yingwei Wang, Ke Ma

**Affiliations:** Pain Management Center and Department of Anesthesiology, Xinhua Hospital, Shanghai Jiaotong University School of Medicine, 1665 Kongjiang Road, Shanghai 200092, China

## Abstract

*Objective*. The aim of this study was to examine the efficacy and safety of pulsed radiofrequency (PRF) in the treatment of chronic migraine (CM) on cervical 2-3 posterior medial branches. *Methods*. This randomized, double-blind, and controlled clinical trial included 40 subjects with CM, who were randomly divided into two groups: treatment (treated by PRF) and sham (treated by sham treatment). Pain intensity, headache duration (days), the Migraine Disability Assessment Questionnaire (MIDAS), and aspirin dose taken by patients were evaluated at 1, 2, and 6 months after the intervention. Side effects were observed from the time of treatment and throughout the follow-up period. *Results*. During the follow-up, pain intensity, headache duration (days), disability score, and the analgesic dose were significantly improved in the treatment group compared to the sham group (*P* < 0.001) and the baseline (*P* < 0.001) at all measured time points after intervention. No serious complications were reported. *Conclusion*. PRF on the cervical 2-3 posterior medial branches could provide satisfactory efficacy in the treatment of CM without obvious adverse effects.

## 1. Introduction

CM is diagnosed in patients who suffer from headache at least 15 days per month or who have at least 8 days per month in which the headaches are associated with symptoms that meet the diagnostic criteria for migraine. Migraine affects approximately 2% of patients worldwide [1]. A high frequency of migraines is associated with an increased risk of neck pain and disability [2]. The overall burdens of migraine are higher than the burdens of epilepsy, stroke, or Parkinson disease [3]. Although numerous medications have been available for patients with migraine, still a few patients are insensitive to these therapies [4, 5]. In addition, the overuse of medicine such as opiates and triptans was one of the most important risks of migraine progression [6, 7]. Therefore, effective invasive treatments on CM could not only relieve the pain but also avoid the possible progression of migraine derived from the medicine overuse.

Recently, occipital nerve stimulation (ONS) has become a novel invasive treatment for primary headaches, including cervicogenic headache, occipital neuralgia, cluster, and migraine [8, 9]. ONS could provide benefits to some patients with CM [4, 10, 11]. However, the incidence of complications with ONS was consistently high in published studies [4, 10]. Lead migration, the most common complication of ONS, occurred in 10–100% patients and always required a second surgery [10, 12]. In addition, it was regarded that the incidence of complication is still high even performed by experienced physicians [13]. The possible mechanism of ONS for CM was based on trigeminal vascular reflection and stimulations on upper cervical nerves could enhance the neurons in afferent dural inputs [14]. Therefore, an optimal invasive therapy for CM should not only target upper cervical nerves but also bring fewer complications.

PRF is a non neurodestructive therapy that has been widely used in treating numerous chronic pain conditions such as postherpetic neuralgia and chronic postoperative pain [15–18]. PRF induced very few complications according to previous studies [16, 19]. However, to the best of our knowledge, few studies have emphasized the ability of PRF in treating CM. Anatomically, cervical 2-3 posterior medial branches are the sources of the third occipital nerves (ONs), which could be a possible target for neuromodulation in CM. Therefore, in this study, we designed a randomized, controlled, and double-blind trial to perform PRF on the cervical 2-3 posterior medial branches in the treatment of CM.

## 2. Methods

### 2.1. Study Participants

The protocol of this clinical trial was approved by the Human Ethics Committee of Xinhua Hospital. Patients at the Pain Center of Xinhua Hospital from Feb. 2012 to Feb. 2014 were considered for inclusion in this study. All patients had clear understanding of the trial and signed consent forms.

### 2.2. Inclusion and Exclusion Criteria

Patients were considered eligible for the study if they met the following inclusion criteria: (1) the patient was older than 18 years of age, (2) the patient had suffered for more than 6 months from CM, (3) CM was diagnosed strictly according to the Third Edition of the International Classification of Headache Disorders (ICHD-III) [20], and (4) the patient experienced a greater than 30% reduction in pain after occipital nerve block (ONB) of the cervical 2-3 posterior medial branches before the trial. The exclusion criteria were as follows: (1) obvious psychosis, (2) inability to follow the advice of the physician, (3) involvement in other trials, (4) pregnancy or trying to conceive, and (5) inability to finish the trial for any other reason.

### 2.3. Grouping, Randomization, and Blinding

**Figure 1:**
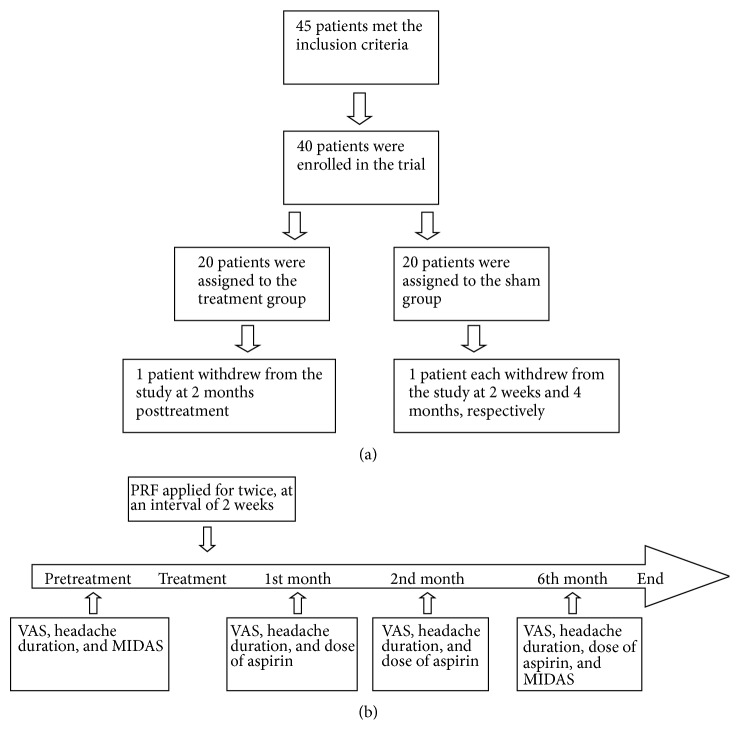
Study flowchart and timeline of the study. (a) Study flowchart. A total of 40 patients were involved in the trial and 37 patients completed the trial. (b) Study timeline described the temporal relationship between the four time points of assessments and the PRF treatments.

Among the 45 patients who met the criteria, 5 patients refused to sign the consent forms. 40 patients were divided into two equal groups by a random number table: a treatment group (treated by PRF) and a sham group (treated with sham treatment). Detailed information on study enrollment and design are shown in the flowchart and timeline (Figure 1). Doctors and patients were blinded to the grouping. Information on grouping was preserved by an investigator who was separated from the operation and follow-up until the end of the trial. There was no communication about the grouping between the investigator who had this information and the investigators related to the clinical trial.

### 2.4. Intervention Procedure

All procedures were performed within a sterile environment with the patient in a prone position. In the first phase, the C-arm machine was placed in the anteroposterior position. The C2 and C3 levels were confirmed by puncture needles through the C-arm. The first entry point was the intersection of the edge of the C2 vertebral bodies and the midline between the C2 and C3 levels. Lidocaine was injected hypodermically to provide local anesthesia. The C-arm machine was changed to the lateral position. A 21-gauge cannula with a 5 mm exposed tip was punctured vertically at C2, as had been marked by the C-arm previously.

**Figure 2:**
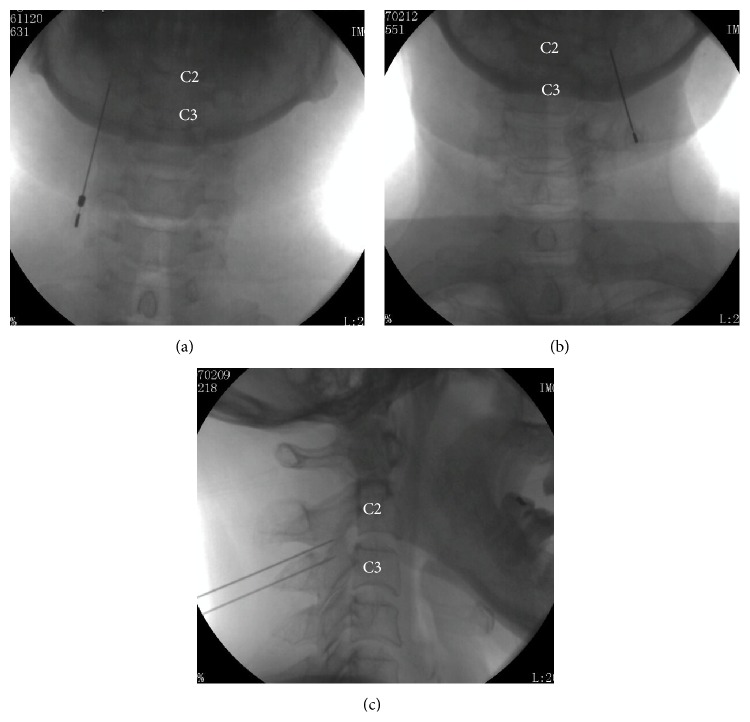
X-ray photos in the PRF treatments. ((a) and (b)) The C-arm machine was placed in the anteroposterior position and the puncture points were at the C2 level. (c) The C-arm machine was placed in the lateral position. The tip of the needles reached the medial branch of C3 and third ONs (C3 and C2, resp.).

The cannula was inserted slowly until the tip reached the front bottom of the C2 inferior articular process, to align with the third ON under the monitoring of the C-arm (Figure 2). The cannula was connected to the radiofrequency generator and the needle tip was adjusted slightly under the sensation test mode (50 HZ, 0.3 V). An abnormal sensation on the part of the patient indicated that the needle was extremely close to the third ON.

The generator was turned to the PRF mode (42°C, 120 seconds, twice for each level). During PRF, the healthcare provider ensured that the cannula did not move. The second entry point was the intersection of the edge of the C3 vertebral body and C3 level. After local anesthesia, the cannula was inserted slowly until the tip reached level of the zygapophyses, to align with the medial branch of C3. The cannula was connected with the generator and the steps were repeated as for the C2 PRF.

In the sham group, the same procedures were applied except that no energy was used. All treatments in both groups were performed unilaterally. The generator was operated by an investigator who was not involved in the follow-up. Patients left the hospital after 1 day of observation. A second PRF or sham treatment was given after an interval of 2 weeks.

### 2.5. Outcome Measures

Follow-up procedures were carried out in 1, 2, and 6 months after the intervention. Pain intensity, headache duration (days), analgesic dose, Migraine Disability Assessment Questionnaire (MIDAS) score, and adverse effects were the main outcome measures that were recorded during the follow-up.

Pain intensity was defined as the average pain intensity during the migraine attack, as recorded on the visual analogue scale (VAS). Pain relief of more than 30% at the 6-month follow-up was defined as “effective.” The headache duration was defined as the number of days that patients suffered from migraine per month. MIDAS was assessed twice: before and 6 months after PRF or sham treatments. Aspirin was used as the routine analgesic, at a dose of 300 mg as needed. The total dose of aspirin used in a month was recorded.

Adverse effects of patients were recorded immediately after the intervention and continued until study completion. In addition to the routine follow-up, patients were able to report the related symptoms to our investigators at the pain clinic. Adverse effects included infection, numbness, increased pain, and paresthesia.

### 2.6. Sample Size and Statistical Analysis

Sample size was calculated by G-power 3.17. Statistical analysis was performed by SPSS19.0. Continuous data were presented as mean ± standard deviation or as the median (interquartile range) if the data were in a skewed distribution. The difference between two groups was calculated by *t*-test. The difference at different time points in the same group was calculated by repeated-measures ANOVA. Differences of enumeration data were evaluated by *χ* ^2^ test.

A sample size calculation was performed to calculate the sample size needed to detect a statistically significant difference at the 0.05 level with a power of 80%. According to a pilot study, pain of patients in the treatment group was reduced by 30 to 40%, compared to 15% in the sham group. Therefore, the calculated minimum total sample size was 36.

## 3. Results

In this study, 40 patients were enrolled and 37 patients completed the follow-up. The demographic characteristics of the patients were similar in both groups. There were no significant differences in sex, age, migraine history, or baseline migraine conditions between the groups (Table 1).

**Figure 3:**
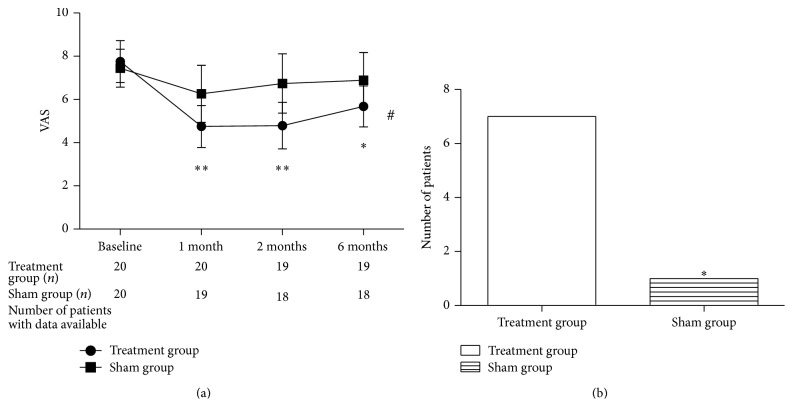
Reduction of pain intensity in the two groups. (a) There was a significant time-related change during the follow-up of the treatment group compared to the sham group. The *P* value of the independent-sample *t*-test refers to the difference between groups in the pain intensity at different time points. The VAS was improved in the first month and stabilized by the sixth month. ^*^ *P* < 0.01 and ^**^ *P* < 0.001 versus the sham group, ^#^ *P* < 0.001 change by time interaction in the treatment group. (b) The histogram demonstrates the number of patients achieving pain reduction. There were significant differences between groups in numbers of the patients achieving more than 30% pain reduction. ^*^ *P* < 0.05 versus the treatment group.

The mean VAS decreased by 2.52 points in the treatment group compared to 0.55 points in the sham group at the 6-month follow-up time point. There was a significant interaction between the variables of treatments and follow-up period (*F* = 111.7, *P* < 0.001). The VAS differed significantly between the treatment and the sham groups at the 1-month (*t* = 4.08, *P* < 0.001), 2-month (*t* = 4.86, *P* < 0.001), and 6-month (*t* = 3.27, *P* < 0.01) follow-up periods. When “effective” was defined as a 30% reduction in pain at the 6-month follow-up, there was a significant difference in the numbers of patients with effective outcomes between the treatment and the sham group (*P* < 0.05). No patient in either group achieved a 50% reduction in pain intensity (Figure 3).

**Figure 4:**
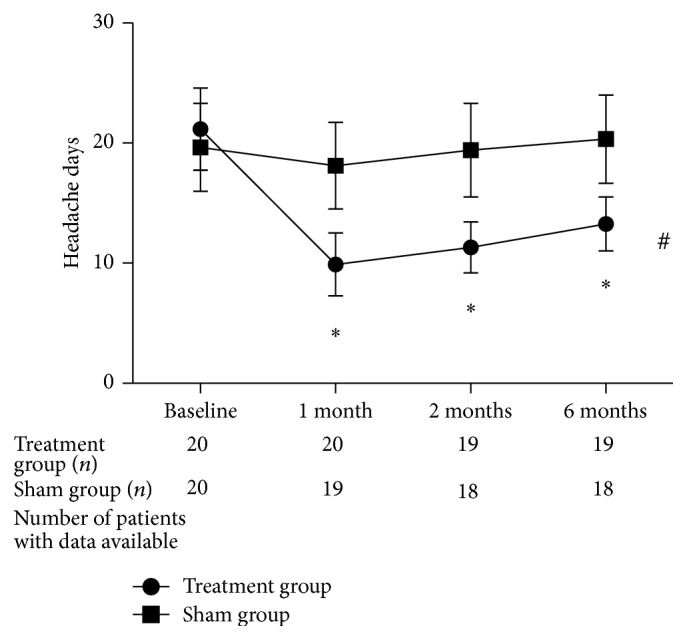
Headache duration in the two groups. Treatments resulted in a significant time-related reduction in the number of days that patients experienced headaches throughout the follow-up period. ^*^ *P* < 0.001 versus the sham group. ^#^ *P* < 0.001 change by time interaction in the treatment group.

The mean decrease of headache duration in the treatment group was 8.9 days per month at the 6-month follow-up. There was a significant interaction between the variables of treatments and follow-up period (*F* = 232.3, *P* < 0.001). There was a significant difference in the decrease of headache duration between the treatment and the sham groups at the 1-month (*t* = 8.14, *P* < 0.001), 2-month (*t* = 7.93, *P* < 0.001), and 6-month (*t* = 7.11, *P* < 0.001) follow-up time points (Figure 4).

**Figure 5:**
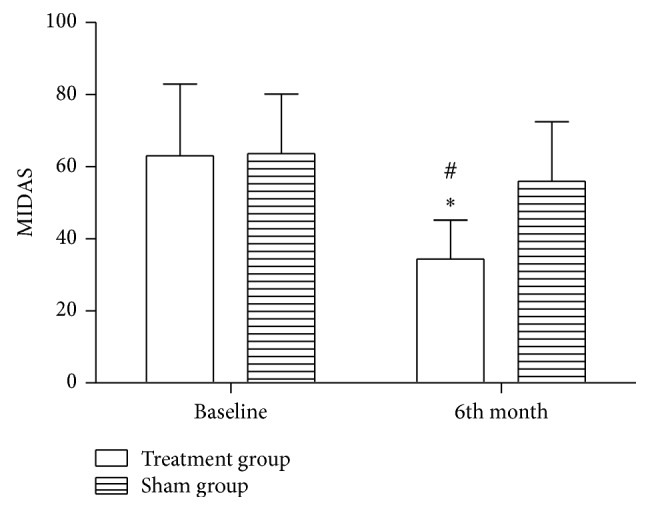
MIDAS scores of the two groups. The MIDAS scores in the treatment group were significantly improved compared to the baseline and to the sham group. ^*^ *P* < 0.001 versus the baseline and ^#^ *P* < 0.001 versus the sham group.

The patients in the treatment group took a significantly lower aspirin dose compared to the patients in the sham group throughout the follow-up period. The aspirin dose differed significantly between these two groups at the 1-month (*t* = 7.0, *P* < 0.001), 2-month (*t* = 6.14, *P* < 0.001), and 6-month (*t* = 6.57, *P* < 0.001) follow-up periods (Table 2). The mean MIDAS score in the treatment group was 21.57 points lower than that in the sham group at the 6-month follow-up time point. The MIDAS scores were significantly decreased after PRF treatment compared to the baseline (*t* = 10.25, *P* < 0.001) and between the two groups (*t* = 4.72, *P* < 0.001, Figure 5).

No patient experienced abnormal bleeding, infection, numbness, postoperative paresthesia, increased pain, or any other complication during the perioperative period. One patient in the treatment group reported mild pain at the injection site after the second round of PRF treatments and the pain subsided within 6 hours without any treatment. No complication was recorded at the follow-up.

## 4. Discussion

In this clinical trial, we have shown that using PRF on the cervical 2-3 posterior medial branches could result in satisfactory efficacy of CM. We chose the cervical 2-3 posterior medial branches as the target in this treatment because of their anatomy. The dorsal ramus of the C2 spinal nerve ultimately becomes the greater ON, which supplies the splenius capitis and semispinalis capitis. The deep branch of the dorsal ramus of the C3 spinal nerve, also known as the third ON, supplies the C2-C3 zygapophyseal joint and the skin over the suboccipital region [9]. The ONs have been regarded as a therapeutic target in migraine. For example, ONB and ONS have been shown to provide benefits in both pain intensity and headache days in migraineurs [1, 21–23]. For these reasons, the cervical 2-3 posterior medial branches were chosen in this clinical trial.

The mechanism of ON-related treatments is mainly based on the trigeminal vascular system [24]. Pain-sensitive structures, including the intracranial vessels, the meninges, and especially the dura mater, are innervated by the ophthalmic ramus of the trigeminal nerve that arises from pseudounipolar neurons located in the trigeminal ganglion. These neurons project onto second-order sensory neurons in the trigeminal nucleus caudalis in the brain stem [25]. The upper cervical roots and nucleus caudalis of the trigeminal tract converge at the C2 level. This convergence is referred to as the trigeminocervical complex.

During migraine, the stimulation of pain-sensitive structures activates neurons of the trigeminal ganglion, which projects to the central nervous system and induces peripheral and central sensitization in migraine [25]. Sensitization of meningeal nociceptors arising from the first-order trigeminal neurons, known as peripheral sensitization, could explain the aggravation of intracranial hypersensitivity in physical activities, such as coughing [26]. Central sensitization is based on the concept that the stimulation of pain-sensitive structures also sensitizes the second-order trigeminovascular neurons located in the medullary dorsal horn (MDH). The MDH receives input from the dura and the periorbital skin, which could explain the hypersensitivity in the periorbital skin [26]. In addition, the cutaneous allodynia is regarded as an individual risk factor for the transformation of CM [1]. Direct stimulation on the ONs could excite the second-order trigeminal afferents in rats [14], which may be the potential mechanism of the therapy of stimulating the cervical 2-3 posterior medial branches in this trial.

Before the PRF treatments, an ONB was applied to help predict the potential curative effect of the target nerves. Similar tests have been performed in former PRF studies [27, 28]. In some ONS studies, ONB was administered to help to provide a clear prediction [10, 12]. However, a recent study found that ONB does not sufficiently predict ONS responsiveness [29]. In our study, approximately 50% of patients achieved a pain reduction of less than 30%, even though they had received a 30% pain reduction after ONB. These results indicated that the nerve block could not provide sufficient prediction of PRF efficacy in CM.

PRF is a minimally invasive neuromodulation approach that has been used to treat chronic pain of various origins [15, 17]. A common working temperature of PRF is 42°C, which is below the minimum threshold for irreversible tissue destruction of 45°C. PRF achieves neuromodulation in numerous aspects, including microstructure damage and the endogenous pathway. In a previous study, microscopic damage was found in the internal ultrastructural components of the axons. This damage was more obvious in C-fibers than in the A-delta or A-beta fibers, consistent with the fact that C-fibers and A-delta fibers are the principal sensory nociceptors [30]. In the endogenous pathway, PRF could increase the level of endogenous opioid precursor mRNA and the corresponding opioid peptide [31]. In a recent study, PRF was able to regulate proinflammatory gene expression at the injury site, dorsal root ganglion (DRG), and spinal cord [32]. These changes along the nociceptive pathway could explain the efficacy of PRF in the peripheral and central aspects of neuropathic pain, such as postherpetic neuralgia [16]. Therefore, in this trial, PRF was utilized to provide neuromodulation of the cervical 2-3 posterior medial branches, to reduce the peripheral and central sensitization of CM and, ultimately, to decrease the pain intensity and headache duration.

No obvious side effects were observed in this trial. PRF is an invasive procedure that provides reversible neuromodulation without tissue damage. The puncture was performed under C-arm monitoring to minimize the risks of injury to the carotid artery or spine. PRF on the cervical 2-3 posterior medial branches has not been reported previously. However, PRF on the greater ON has been used in the treatment of cervicogenic headache and no serious complications were reported [28]. This finding indicated that the greater ON (cervical 2-3 posterior medial branches) was a safe therapeutic target for chronic headache. Compared to the numerous complications that were associated with ONS, including lead migration and infection [10, 13], PRF on the cervical 2-3 posterior medial branches was easier to perform and associated with fewer complications. In addition, PRF was a minimally invasive therapy that led to less treatment-related pain compared to ONS.

There were some limitations in this clinical trial. The trial was designed as a single-center study and had a small sample size. The follow-up period was only 6 months. Therefore, the long-term efficacy of this therapy could not be determined. Moreover, it remained unknown whether the efficacy of PRF on the cervical 2-3 posterior medial branches is superior to ONS. These limitations could be addressed with future studies.

## 5. Conclusion

PRF on the cervical 2-3 posterior medial branches could provide a satisfactory treatment that can reduce pain intensity, headache duration, and disability scores. The procedure was relatively easy to perform and resulted in few side effects.

## Figures and Tables

**Table 1:** Patients' demographics and baseline headache data.

Group	Treatment	Sham
Men/women (number of patients)	3/17	4/16
Age (years)	43.5 ± 11.07	43.55 ± 7.82
Headache history (years)	15.25 ± 8.37	18.75 ± 9.98
Baseline VAS	7.75 ± 0.96	7.45 ± 0.88
Baseline headache duration (days/month)	21.05 ± 3.36	19.65 ± 3.66
Baseline MIDAS score	63.05 ± 19.89	63.60 ± 16.59

There was no significant difference between two groups in these data above.

**Table 2:** The mean doses of aspirin taken by patients in the treatment group were significantly lower than those in the sham group.

Group	1st month	2nd month	6th month
Treatment	6.15 ± 2.03^*^ (*n* = 20)	6.16 ± 2.58^*^ (*n* = 19)	6.37 ± 1.83^*^ (*n* = 19)
Sham	14.79 ± 5.10 (*n* = 19)	14.32 ± 5.17 (*n* = 18)	14.00 ± 4.71 (*n* = 18)

^*^ *P* < 0.001 versus the sham group.

## References

[1] Schwedt T. J. (2014). Chronic migraine. *British Medical Journal*.

[2] Florencio L. L., Chaves T. C., Carvalho G. F. (2014). Neck pain disability is related to the frequency of migraine attacks: a cross-sectional study. *Headache*.

[3] Leonardi M. (2014). Higher burden of migraine compared to other neurological conditions: results from a cross-sectional study. *Neurological Sciences*.

[4] Silberstein S. D., Dodick D. W., Saper J. (2012). Safety and efficacy of peripheral nerve stimulation of the occipital nerves for the management of chronic migraine: results from a randomized, multicenter, double-blinded, controlled study. *Cephalalgia*.

[5] Fu C., Yu L., Zou Y. (2012). Efficacy of chuanxiong ding tong herbal formula granule in the treatment and prophylactic of migraine patients: a randomized, double-blind, multicenter, placebo-controlled trial. *Evidence-Based Complementary and Alternative Medicine*.

[6] Bigal M. E., Lipton R. B. (2008). Excessive acute migraine medication use and migraine progression. *Neurology*.

[7] Dougherty C., Silberstein S. D. (2014). Providing care for patients with chronic migraine: diagnosis, treatment, and management. *Pain Practice*.

[8] Perini F., de Boni A. (2012). Peripheral neuromodulation in chronic migraine. *Neurological Sciences*.

[9] Jasper J. F., Hayek S. M. (2008). Implanted occipital nerve stimulators. *Pain Physician*.

[10] Saper J. R., Dodick D. W., Silberstein S. D., McCarville S., Sun M., Goadsby P. J. (2011). Occipital nerve stimulation for the treatment of intractable chronic migraine headache: ONSTIM Feasibility Study. *Cephalalgia*.

[11] Notaro P., Buratti E., Meroni A., Montagna M. C., Rubino F. G., Voltolini A. (2014). The effects of peripheral occipital nerve stimulation for the treatment of patients suffering from chronic migraine: a single center experience. *Pain Physician*.

[12] Schwedt T. J., Dodick D. W., Hentz J., Trentman T. L., Zimmerman R. S. (2007). Occipital nerve stimulation for chronic headache—long-term safety and efficacy. *Cephalalgia*.

[13] Diener H.-C. (2012). Occipital nerve stimulation for chronic migraine: already advised?. *Cephalalgia*.

[14] Bartsch T., Goadsby P. J. (2002). Stimulation of the greater occipital nerve induces increased central excitability of dural afferent input. *Brain*.

[15] Snidvongs S., Mehta V. (2010). Pulsed radio frequency: a non-neurodestructive therapy in pain management. *Current Opinion in Supportive and Palliative Care*.

[16] Ke M., Yinghui F., Yi J. (2013). Efficacy of pulsed radiofrequency in the treatment of thoracic postherpetic neuralgia from the angulus costae: a randomized, double-blinded, controlled trial. *Pain Physician*.

[17] Yang Y., Dai L., Ma K. (2015). Spontaneous muscle contraction with extreme pain after thoracotomy treated by pulsed radiofrequency. *Pain Physician*.

[18] Taverner M., Loughnan T. (2014). Transcutaneous pulsed radiofrequency treatment for patients with shoulder pain booked for surgery: a double-blind, randomized controlled trial. *Pain Practice*.

[19] Vigneri S., Sindaco G., Gallo G. (2014). Effectiveness of pulsed radiofrequency with multifunctional epidural electrode in chronic lumbosacral radicular pain with neuropathic features. *Pain Physician*.

[20] Hui J., Zhang Z.-J., Zhang X., Shen Y., Gao Y.-J. (2013). Repetitive hyperbaric oxygen treatment attenuates complete Freund's adjuvant-induced pain and reduces glia-mediated neuroinflammation in the spinal cord. *The Journal of Pain*.

[21] Ashkenazi A., Matro R., Shaw J. W., Abbas M. A., Silberstein S. D. (2008). Greater occipital nerve block using local anaesthetics alone or with triamcinolone for transformed migraine: a randomised comparative study. *Journal of Neurology, Neurosurgery and Psychiatry*.

[22] Takmaz S. A., Inan N., Üçler S., Yazar M. A., Inan L., Başar H. (2008). Greater occipital nevre block in migraine headache: preliminary results of 10 patients. *Ağrı*.

[23] Afridi S. K., Shields K. G., Bhola R., Goadsby P. J. (2006). Greater occipital nerve injection in primary headache syndromes—prolonged effects from a single injection. *Pain*.

[24] Goadsby P. J., Hargreaves R. (2008). Refractory migraine and chronic migraine: pathophysiological mechanisms. *Headache*.

[25] Link A. S., Kuris A., Edvinsson L. (2008). Treatment of migraine attacks based on the interaction with the trigemino-cerebrovascular system. *The Journal of Headache and Pain*.

[26] Bernstein C., Burstein R. (2012). Sensitization of the trigeminovascular pathway: perspective and implications to migraine pathophysiology. *Journal of Clinical Neurology*.

[27] Zhang J., Shi D.-S., Wang R. (2011). Pulsed radiofrequency of the second cervical ganglion (C2) for the treatment of cervicogenic headache. *Journal of Headache and Pain*.

[28] Gabrhelík T., Michálek P., Adamus M. (2011). Pulsed radiofrequency therapy versus greater occipital nerve block in the management of refractory cervicogenic headache—a pilot study. *Prague Medical Report*.

[29] Kinfe T. M., Schuss P., Vatter H. (2014). Occipital nerve block prior to occipital nerve stimulation for refractory chronic migraine and chronic cluster headache: myth or prediction?. *Cephalalgia*.

[30] Erdine S., Bilir A., Cosman E. R., Cosman E. R. (2009). Ultrastructural changes in axons following exposure to pulsed radiofrequency fields. *Pain Practice*.

[31] Moffett J., Fray L. M., Kubat N. J. (2012). Activation of endogenous opioid gene expression in human keratinocytes and fibroblasts by pulsed radiofrequency energy fields. *Journal of Pain Research*.

[32] Vallejo R., Tilley D. M., Williams J., Labak S., Aliaga L., Benyamin R. M. (2013). Pulsed radiofrequency modulates pain regulatory gene expression along the nociceptive pathway. *Pain Physician*.

